# Predicting glycemic control status and high blood glucose levels through voice characteristic analysis in patients with cystic fibrosis-related diabetes (CFRD)

**DOI:** 10.1038/s41598-023-35416-w

**Published:** 2023-05-27

**Authors:** Pichatorn Suppakitjanusant, Nittaya Kasemkosin, Alisa K. Sivapiromrat, Samuel Weinstein, Boonsong Ongphiphadhanakul, William R. Hunt, Viranuj Sueblinvong, Vin Tangpricha

**Affiliations:** 1 Chakri Naruebodindra Medical Institute, Faculty of Medicine Ramathibodi Hospital, Mahidol University, Bangkok, Samut Prakan Thailand; 2 Division of Endocrinology, Metabolism and Lipids, Department of Medicine, Emory University School of Medicine, Atlanta, USA; 3 Department of Communication Science and Disorders, Faculty of Medicine Ramathibodi Hospital, Mahidol University, Bangkok, Thailand; 4 Emory College of Arts and Sciences, Emory University, Atlanta, USA; 5 Division of Endocrinology and Metabolism, Department of Medicine, Faculty of Medicine Ramathibodi Hospital, Mahidol University, Bangkok, Thailand; 6 Division of Pulmonary, Allergy, Critical Care, and Sleep Medicine, Department of Medicine, Emory University School of Medicine, Atlanta, USA

**Keywords:** Diabetes, Cystic fibrosis, Biomedical engineering

## Abstract

Cystic fibrosis-related diabetes (CFRD) is associated with reduced life expectancy in adults with cystic fibrosis (CF). Voice analysis may be a convenient method for diagnosing and monitoring CFRD. This study aims to determine the relationship between voice characteristics and markers of glucose and glycemic control and to identify if voice analysis can predict high blood glucose levels and glycemic control in adults with CFRD. We conducted a prospective cross-sectional study in adults with CF from March to December 2021. We recorded 3-second voice samples of a sustained /a/ vowel and analyzed voice characteristic using the Computerized Speech Lab with the Multi-Dimensional Voice Program. In female participants with CFRD, the noise-to-harmonic ratio was significantly lower in those with HbA1c ≥ 7. Furthermore, fundamental frequency variation was significantly lower in both male and female participants with CFRD who had a glucose level of 200 mg/dL or higher at the time of collection. This finding was also associated with a high level of point-of-care glucose. The human voice has potential as a non-invasive tool for measuring glucose levels and glycemic control status in CFRD patients in the future.

## Introduction

Cystic fibrosis (CF)-related diabetes (CFRD) is the most common non-pulmonary comorbidity in CF with almost 50% of adults with CF developing CFRD during their lifetime^1^. The development of CFRD is associated with worse nutritional status, increased risk of microvascular complications, decreased pulmonary function, and decreased overall physical health, leading to reduced life expectancy in adults with CF^2^. Patients with CFRD are typically asymptomatic for years prior to diagnosis, resulting in a lack of early treatment and a potentially increased risk of complications. Earlier diagnosis CFRD in adults with CF may avoid complications and decrease mortality^2^.

The CF Foundation recommends annual screening for CFRD annually with the oral glucose tolerance test (OGTT)^3^. During the COVID-19 pandemic, most CF clinics utilized telemedicine to mitigate the risk of contracting COVID-19, resulting in challenges in performing blood tests to measure glucose and hemoglobin A1c (HbA1c) concentrations^4^. Although 75% of U.S. youth and adults with CFRD reported continuous glucose monitoring use^5^, there are some barriers including the cost of the device and supplies, insurance coverage, and complexity of the device^6^. Despite the increase in non-invasive glucose monitoring devices, more convenient methods of diagnosing and monitoring CFRD are necessary to prevent complications for adults with CF.

Changes in blood glucose levels may cause changes in the elastic properties of laryngeal soft tissue, resulting in changes in the tissue compliance based on Hooke’s law of physics^7^. Studies in voice acoustic analysis have demonstrated significant differences in fundamental frequency, jitter, shimmer, and noise-to-harmonic ratio when comparing healthy and patients with diabetes^8,9^. Neurological, vascular, and muscular changes are associated with impaired glycemic control. Prior studies indicated differences in voice parameters among adults with CF who have and have not been diagnosed with CFRD^10,11^. Thus, voice acoustic analysis may potentially be used to screen for CFRD screening and to monitor home glucose values. The effects of high blood glucose concentrations on voice characteristics in patients with CFRD are poorly understood.

This study aims to determine the relationship between voice characteristics and markers of glucose and glycemic control. The secondary goal is to determine if voice analysis can predict high blood glucose levels and glycemic control.

## Methods

### Study participants

This was a prospective pilot study with CF at the Emory Adult CF Center recruited between March and December 2021. The study was approved by the Emory University Institutional Review Board. All participants provided signed informed consent before participating in the study. The inclusion criteria included adults with CF between the age of 18 and 70 years, a diagnosis of CF based on CFTR gene mutation or sweat chloride testing, and a diagnosis of cystic fibrosis-related diabetes (CFRD) based on a standard OGTT. The upper limit of 70 years was determined based on voice changes found in the elderly^12^. Exclusion criteria included pregnancy, breastfeeding, uncontrolled hypertension (systolic blood pressure > 140 mmHg or diastolic blood pressure > 90 mmHg), acute myocardial infarction or stroke within 6 months, history of thyroid disease, history of substance abuse, neurological disorder, active mental disorders, active smoking or former smokers who had quit smoking less than 6 months, speech disorders, history of abnormal voice such as hoarseness of voice, upper respiratory tract infection and acute pulmonary exacerbation within the previous 2 weeks. We excluded individuals with uncontrolled hypertension due to the potential change in voice parameters^13,14^ and individuals with a history of thyroid disease due to potential laryngeal apparatus distortion^15^.

### Study protocol

Participants who met the eligibility criteria were interviewed using case record forms to collect demographic data and the duration of the disease. Relevant medical information, including results from the OGTT, hemoglobin A1c and serum glucose, was extracted from the medical record and from the participant questionnaire. Glycemic control was defined as the HbA1c level using 7% as a cutoff point on the day of the voice recording or within the previous three months.

### Glucose measurements

Point-of-care blood glucose testing (POCT) was conducted at the participant’s clinic visit using the OneTouch Verio Flex glucometer (Malvern, Pennsylvania). Capillary glucose measurement was performed by pricking a sterile lancet on one of the participant’s fingertips with a sterile lancet to obtain a drop of blood for use in the glucometer.

### Voice recordings

During an outpatient clinic appointment, the participant was requested to produce a sustained vowel, /a:/, at their usual frequency and strength after taking a deep breath, in order to obtain maximum phonation time without using expiratory reserve air. Patients were seated in a soundproof room with a head-mounted mouth microphone positioned 4 cm from the lips and 45 to 90 degrees from the front of the mouth. All voice datasets were amplified and analog-to-digital conversed via USBPre2 (Sound Devices LLC, Reedsburg, Wisconsin). Using voice recorder software (Apple, Cupertino, California), voice samples are saved as .wav files. An initial 3.5 s recording of the vowel /a:/ emission was collected for voice acoustic analysis; the commencement of emission was omitted to prevent interference from voice attachment on data processing.

### Voice acoustic assessment

Acoustic parameters were analyzed using Computerized Speech Lab (CS) model 4500 (PENTAX Medical, New Jersey, USA). CSL is considered the gold standard system for acoustic analysis and has been validated to assess voice pathology in many controlled trials^9,16^. The Multi-Dimensional Voice Program (MDVP), a computerized voice analysis system, in conjunction with CSL, is a versatile voice-processing and spectrographic analysis software package ideally suited for use in determining acoustic parameters including fundamental frequency (F0), fundamental frequency variation (vF0), jitter, shimmer, relative average perturbation (RAP), and noise-to-harmonic ratio (NHR).

F0, measured in Hertz (Hz), is defined as the number of times a sound wave produced by the vocal cords repeats during a given time period. It is also the number of cycles of opening/closure of the glottis. vF0, measured in %, represents the relative standard deviation of the period-to-period calculated F0.

Jitter, measured in %, refers to frequency variation from cycle to cycle. It is affected mainly by the lack of control of the vibration of the vocal cords. Shimmer, measured in %, relates to the amplitude variation of the sound wave. It changes due to the reduction of glottal resistance and mass lesions on the vocal cords and is correlated with the presence of noise emission and breathiness^17^.

RAP, measured in %, is a quantitative measure of the voice while producing a sustained vowel. It minimizes the effects of slow changes in the F0 by averaging across three successive vibratory cycles to provide an estimate of the value of the middle period had there been no perturbation present^17^. NHR is a general evaluation of noise present in vocalization. It is an assessment of the ratio between the non-periodic components, which are the glottal noise, and periodic components, which are the vibration of the vocal cords. The quality of the recording of each patient’s voice, namely the sustained /a:/, was blindly evaluated by a speech therapist (NK).

### Statistical analysis

All study data were checked for normality and presented as mean (SD) if they were normally distributed, or median (interquartile range, IQR) if they were not normally distributed. For baseline characteristic data in Table 1, the independent *t* test or Mann–Whitney *U* test was used to compare all continuous variables and the Chi-square test was used to compare categorical variables between the two groups. Multivariate analysis and logistic regression analysis were performed to identify independent variables associated with CFRD. A two-tailed p-value of less than 0.05 was considered statistically significant. Statistical analyses were performed using Stata version 15 (StataCorp LLC, College Station, Texas).

**Table 1:** Baseline characteristics of patients with CFRD.

Variables	CFRD with HbA1c < 7% N = 25	CFRD with HbA1c ≥ 7% N = 18	*p* value
Age (years)	36 ± 14	32 ± 13	0.269
Height (cm)	164.60 ± 9.89	164 ± 12.35	0.959
Weight (kg)	65.76 ± 13.56	62 ± 19.87	0.536
BMI (kg/m^2^)	24.15 ± 3.83	23 ± 7.17	0.466
Diabetes profiles			
HbA1c level (%)	5.9 ± 0.4	9 ± 2.2	**< 0.01**
POCT glucose level (mg/dl)	123 ± 33	183 ± 87	0.012
Duration of CFRD diagnosis (years)	8 ± 7	10 ± 7	0.279
Race			0.071
Caucasian	22 (88%)	14 (77.78%)	
Non-Caucasian	3 (12%)	4 (22.22%)	
Sex			0.147
Male	14 (56%)	11 (61.11%)	
Female	11 (44%)	7 (38.89%)	
Smoking status			0.144
Never smoked	23 (92%)	16 (88.89%)	
Former smoker	2 (8%)	2 (11.11%)	

Significant values are in bold.

## Results

### Demographics of study participants

A total of 43 participants were recruited in this study. There were 25 participants with CFRD who had an HbA1c < 7% and 18 participants with CFRD who had an HbA1c ≥ 7%. Baseline characteristics are presented in Table 1. There were no significant differences in the participant demographics between the two groups. The participants with CFRD who were classified in the group of HbA1c < 7% had a mean HbA1c level of 5.9 ± 0.4% and a mean POCT glucose level of 123 ± 33 mg/dL. As expected, the participants with CFRD who were classified in the group of HbA1c ≥ 7% had a mean HbA1c level of 9 ± 2.2% and a mean POCT glucose level of 183 ± 87 mg/dL.

### Voice characteristics as a marker of glycemic control

When stratified by sex in Table 2, female participants with CFRD who had an HbA1c ≥ 7% had a significantly lower NHR than female participants with CFRD had an HbA1c < 7% (0.13 ± 0.02 vs 0.15 ± 0.03, *p* = 0.049). However, there was no significant difference in male participants with CFRD regarding glycemic control status. We performed multivariate linear regression analysis in Table 3 to determine NHR as a dependent variable, was significantly associated with glycemic control (*p* = 0.041) and female sex (*p* = 0.047). NHR decreased by 0.0058 for every 1% increase in HbA1c in participant with CFRD. NHR is expected to be 0.0218 lower in female individuals compared to male individuals. Independent variables included age, BMI, sex, duration of CFRD diagnosis, POCT glucose level, and HbA1c level. To assess NHR as a tool for the prediction of glycemic control status, we performed a logistic regression analysis with HbA1c ≥ 7% as an outcome and subject characteristics including age, BMI, sex, and duration of CFRD diagnosis as relevant variables in Table 4. Results showed that NHR did not significantly predict CFRD participants with HbA1c ≥ 7% when other factors were taken into account (*p* = 0.168).

**Table 2:** Mean and standard deviation of acoustic parameter among cystic fibrosis-related diabetes patients who have HbA1c ≤ 7% and those who have HbA1c > 7%, categorized by sex (male/female).

Acoustic parameters	Male (n = 25)			Female (n = 18)		
	HbA1c < 7% (n = 14)	HbA1c ≥ 7% (n = 11)	*p *value	HbA1c < 7% (n = 11)	HbA1c ≥ 7% (n = 7)	*p *value
F0 (Hz)	133.93 ± 24.32	136.80 ± 50.87	0.865	213.45 ± 52.88	228.70 ± 38.16	0.488
vF0 (%)	1.56 ± 0.64	1.41 ± 0.74	0.602	1.28 ± 0.55	0.95 ± 0.37	0.958
Jitter (%)	1.25 ± 0.75	1.10 ± 0.69	0.610	1.00 ± 0.77	0.73 ± 0.65	0.424
Shimmer (%)	6.55 ± 3.84	6.23 ± 2.12	0.798	4.86 ± 2.30	3.95 ± 1.01	0.269
NHR	0.17 ± 0.05	0.16 ± 0.03	0.762	**0.15 ± 0.03**	**0.13 ± 0.02**	**0.049**
VTI	0.08 ± 0.02	0.07 ± 0.04	0.689	0.06 ± 0.01	0.06 ± 0.02	0.951
RAP (%)	0.75 ± 0.45	0.65 ± 0.42	0.579	0.63 ± 0.45	0.43 ± 0.40	0.338

Significant values are in bold.

**Table 3:** Multivariate linear regression analysis with noise-to-harmonic Ratio (NHR) as an outcome.

Variables	Noise-to-harmonic ratio (NHR)	
	Coefficients	*p* value
Age	0.0002	0.574
BMI	− 0.0011	0.329
Female	− **0.0218**	**0.047**
Duration of CFRD diagnosis	0.0003	0.667
Glucose level	0.0001	0.200
HbA1c level	− **0.0058**	**0.041**
Adjusted R^2^	0.0970	

Significant values are in bold.

**Table 4:** Logistic regression analysis of noise-to-harmonic Ratio (NHR) and relevant variables in participants with CFRD and HbA1c ≥ 7%.

Variables	CFRD with HbA1c ≥ 7%	
	Coefficients	*p* value
Age	− 0.062	0.108
BMI	− 0.021	0.810
Female	− 0.567	0.443
Former smoking	1.955	0.226
Duration of CFRD diagnosis	0.081	0.127
NHR	− 15.887	0.168
Chi Square	7.0431	

### Voice characteristics as a marker of high glucose level

When analyzing CFRD participants with a POCT glucose level ≥ 200 mg/dL versus CFRD participants with a POCT glucose level < 200 mg/dL in Table 5, vF0 was significantly lower in both males (1.14 ± 0.14 vs 1.58 ± 0.73, *p* = 0.017) and female participants (0.75 ± 0.07 vs 1.26 ± 0.56, *p* = 0.002). Moreover, there was a significantly lower RAP in female participants with CFRD who have POCT glucose level ≥ 200 mg/dL as compared to those who have POCT glucose level < 200 mg/dL (0.31 ± 0.13 vs 0.66 ± 0.50, *p* = 0.040). To determine vF0 and RAP as dependent variables significantly associated with glucose level, we conducted multivariate linear regression analysis in Table 6. Data showed that the duration of CFRD and POCT glucose levels were statistically related to vF0, *p* = 0.019 and *p* = 0.012 respectively. However, in RAP, there was no significant association with POCT glucose level. We performed logistic regression analysis with POCT glucose level ≥ 200 mg/dL as an outcome in Table 7, to assess vF0 as a tool for the prediction of high glucose levels. Results demonstrated that a decrease of 1% vF0 is significantly associated with an increase of 1.2% in the odds of POCT glucose level ≥ 200 mg/dL (*p* = 0.028) and the longer duration of 1 year CFRD significantly associated with an increase of 19.12% in the odds of POCT glucose level ≥ 200 mg/dL (*p* = 0.042).

**Table 5:** Mean and standard deviation of acoustic parameter among cystic fibrosis-related diabetes patients who have POCT glucose level ≥ 200 mg/dL and those who have POCT glucose level < 200 mg/dL, categorized by sex (Male/ Female).

Acoustic parameters	Male (n = 25)			Female (n = 18)		
	POCT glucose level < 200 mg/dL (n = 20)	POCT glucose level ≥ 200 mg/dL (n = 5)	*p *value	POCT glucose level < 200 mg/dL (n = 14)	POCT glucose level ≥ 200 mg/dL (n = 4)	*p *value
F0 (Hz)	132.58 ± 24.02	150.69 ± 56.14	0.493	211.39 ± 58.29	231.40 ± 33.25	0.493
vF0 (%)	**1.58 ± 0.73**	**1.14 ± 0.14**	**0.017**	**1.26 ± 0.56**	**0.75 ± 0.07**	**0.002**
Jitter (%)	1.24 ± 0.77	0.97 ± 0.36	0.271	1.04 ± 0.85	0.52 ± 0.21	0.053
Shimmer (%)	6.48 ± 3.33	6.15 ± 2.56	0.818	4.85 ± 2.45	4.19 ± 1.06	0.613
NHR	0.17 ± 0.04	0.17 ± 0.03	0.991	0.15 ± 0.02	0.13 ± 0.01	0.294
VTI	0.07 ± 0.03	0.08 ± 0.02	0.738	0.06 ± 0.02	0.06 ± 0.01	0.614
RAP (%)	0.74 ± 0.47	0.56 ± 0.24	0.264	**0.66 ± 0.50**	**0.31 ± 0.13**	**0.040**

Significant values are in bold.

**Table 6:** Multivariate linear regression analysis with fundamental frequency variation (vF0) and relative average perturbation (RAP) as outcomes. Significant values are in bold.

Variables	Fundamental frequency variation (vF0)		Relative average perturbation (RAP)	
	Coefficients	*p* value	Coefficients	*p* value
Age	0.013	0.065	0.008	0.142
BMI	− 0.016	0.348	− 0.025	0.062
Female	− 0.227	0.180	− 0.085	0.517
Duration of CFRD diagnosis	**0.030**	**0.019**	0.005	0.571
POCT glucose level	− **0.003**	**0.012**	− 0.002	0.083

**Table 7:** Logistic regression analysis of fundamental frequency variation (vF0) and relevant variables on high glucose levels (POCT glucose level ≥ 200 mg/dL).

Variables	High glucose levels (POCT glucose level ≥ 200 mg/dL)	
	Coefficients	*p* value
Age	− 0.021	0.653
BMI	− 0.037	0.764
Female	− 1.075	0.348
Duration of CFRD diagnosis	0.175	**0.028**
vF0	− 4.392	**0.042**

Adjusted R^2^ of the logistic regression = 14.507. Significant values are in bold.

## Discussion

The principal goal of this study was to determine whether voice parameters differed by glycemic control status and high blood glucose level in people with CFRD. We found that, in female participants with CFRD, NHR was significantly lower in those who had an HbA1c ≥ 7%. NHR was also associated with HbA1c level when adjusted for age, BMI, duration of diabetes diagnosis, and glucose level. In addition, vF0 was significantly lower in both male and female participants with CFRD who had a glucose level of 200 mg/dL or higher at the time of collection. The vF0 value was also associated with a high level of point-of-care glucose. Other voice parameter changes including F0, Jitter, Shimmer and VTI were not significantly correlated with HbA1c level and glucose level in adults with CFRD.

There are a number of potential mechanisms for the differences in NHR in females with CFRD regarding their HbA1c level. NHR has been reported to assess vocal fold behavior^17^. Studies have shown diabetes-related decrements in laryngeal function in the phonatory apparatus in diseases such as myopathy and neuropathy^18,19^. Glycemic status may lower NHR by contributing to instability in vocal fold vibration and thus interfering with the periodicity of vibration^20^. Changes in voice parameters may be more prominent in females compared to males with CFRD. There is no clear mechanism why females with CFRD have prominent changes in voice parameters compared to males. A study in adults without CF demonstrated that laryngeal geometry, hormone differences, and behavior characteristics of women were prone to have more voice disorders than men^21^. Changes in voice characteristics including vF0 and RAP mainly reflect laryngeal muscle behavior which differs between sexes. vF0 has been used to measure the ability to keep the laryngeal muscles in a fixed position for vowel prolongation^22^ while RAP has been used to measure the intrinsic stability of the voice in terms of contraction of laryngeal muscles during sound production^17^. High glucose levels may decrease the strength of intrinsic laryngeal muscles can result in a decrease in vF0 and RAP in our study. Moreover, poor nutrition due to malabsorption in CF may cause decreased laryngeal muscle mass leading to laryngopharyngeal tract hypomobility. Our study did not find any association between BMI and HbA1c level well as BMI and glucose level in participants with CFRD. Although BMI  grossly represents nutritional status, the measurement for laryngeal muscle mass needs to be examined in future studies to confirm this hypothesis.

Changes in other voice characteristics have been studied in participants with CF. One particular characteristic is the voice turbulence index (VTI)^17^ which measures the relative energy level of high-frequency noise. This characteristic correlates with the turbulence caused by incomplete or loose adduction of the vocal folds and clinically correlates in patients with laryngopharyngeal reflux^23^. Our prior study focused on detecting CFRD using changes in voice characteristics and showed significantly higher VTI in males with CFRD compared to male CF patients without CFRD^10^ whereas this study demonstrated that there is no significant difference of VTI in individuals with CFRD according to glycemic control status or POCT glucose level. This can be explained by gastrointestinal acid reflux in individuals with CFRD leading to laryngopharyngeal reflux^24^. However, there is no specific sex differences in prevalence of acid reflex endoscopy-based study in CFRD.

The number of studies on the voice characteristic analysis in non-CF diabetes is limited. Three studies in adults with type 2 diabetes had mixed results. A study in India demonstrated a reduction of NHR, RAP and shimmer in participants with diabetes compared to participants without diabetes^8^. A study in Thailand showed significantly lowered F0 in females with diabetes and no change was detected in RAP as compared to females without diabetes^9^ However, the study in Turkey found a significantly higher of absolute Jitter in participants with diabetes compare to healthy volunteer. There is also a significant lowering maximum phonation time in individuals with diabetes neuropathy^25^. There is only one study in pediatric individuals between 6 and 18 years that found no significant difference in acoustic voice quality index in children with type 1 diabetes compared to age and sex-matched healthy participants^26^. Further study on voice characteristic analysis are needed to understand more about effect of diabetes in voice.

Developing this new voice technology may improve screening for CFRD. One advantage of this technology is that glycemic control can be indirectly associated with the voice as a non-invasive technique. Although not studied directly in this study, future applications could involve the use of this technology to approximate real-time markers of glycemic control without requiring an invasive procedure such as a finger prick. Other potential applications include monitoring of long-term CFRD glycemic status, which would save the time in clinic and/or the need for venipuncture for a patient. Also, this technique can be used in telehealth visits, which further provides another potential use of this technology.

This study of voice characteristic analysis provides an initial step in the understanding of the physiology of voice changes occurring in patients with CFRD. Several studies show voice signals have the potential to be used to predict abnormal blood glucose levels in non-CF diabetes^27,28^. Our previous study in deep learning-based voice technique for CFRD screening demonstrated the potential of employing artificial intelligence to analyze this data^29^. Future directions of this work include utilizing artificial intelligence methods, developing a mobile application for monitoring glucose levels and long-term glycemic control in CFRD, and assessing the technological feasibility and the cost of the equipment.

## Limitations

Our study provides important data regarding the relationship between voice characteristics and glycemic control in adults with CF; however, there are a few limitations. One limitation is the small number of samples in the different glycemic control categories which may not enough to detect the difference between the range of HbA1c level Future studies need to have a larger number of subjects in different categories of glycemic control levels to have sufficient statistical power to determine the relationship with various voice characteristics. Our study may not be generalizable to other forms of diabetes as we only focused on CFRD. Another limitation is the lack of laryngeal imaging which would be useful in interpreting the results of the study. To better understand the functionality of vocal folds, a laryngeal imaging technique like videostroboscopy could be a part of a future study. Finally, the study was done in a soundproof room under extremely controlled conditions. Future studies need to focus on voice recordings performed in an ambient environment to develop the practical clinical application for this technology.

## Conclusions

We found differences in voice parameters that distinguished normal glycemic control from inadequate and poorly controlled CFRD in patients with CF. There is potential to use the human voice as a non-invasive tool for glycemic control assessment in patients with CFRD. NHR was significantly associated with poor glycemic control status while vF0 was associated with high point-of-care glucose levels in participants with CFRD. Larger sample sizes examining voice characteristics and laryngeal imaging techniques are warranted to further investigate the feasibility of using voice as a biomarker for glycemic control in CFRD.

## Data Availability

The datasets generated during and/or analyzed during the current study are not publicly available due to health privacy and information security but are available from the corresponding author on reasonable request.
