# Author Correction: Predicting glycemic control status and high blood glucose levels through voice characteristic analysis in patients with cystic fibrosis-related diabetes (CFRD)

**DOI:** 10.1038/s41598-023-37792-9

**Published:** 2023-07-03

**Authors:** Pichatorn Suppakitjanusant, Nittaya Kasemkosin, Alisa K. Sivapiromrat, Samuel Weinstein, Boonsong Ongphiphadhanakul, William R. Hunt, Viranuj Sueblinvong, Vin Tangpricha

**Affiliations:** 1 Chakri Naruebodindra Medical Institute, Faculty of Medicine Ramathibodi Hospital, Mahidol University, Bangkok, Samut Prakan Thailand; 2 Division of Endocrinology, Metabolism and Lipids, Department of Medicine, Emory University School of Medicine, Atlanta, USA; 3 Department of Communication Science and Disorders, Faculty of Medicine Ramathibodi Hospital, Mahidol University, Bangkok, Thailand; 4 Emory College of Arts and Sciences, Emory University, Atlanta, USA; 5 Division of Endocrinology and Metabolism, Department of Medicine, Faculty of Medicine Ramathibodi Hospital, Mahidol University, Bangkok, Thailand; 6 Division of Pulmonary, Allergy, Critical Care, and Sleep Medicine, Department of Medicine, Emory University School of Medicine, Atlanta, USA

Correction to: *Scientific Reports* https://doi.org/10.1038/s41598-023-35416-w, published online 27 May 2023

The original version of this Article contained an error in the spelling of the author Samuel Weinstein which was incorrectly given as Samuel Weinsein.

The original Article has been corrected.

